# Hyperpigmentation Inhibits Early Skeletal Muscle Development in Tengchong Snow Chicken Breed

**DOI:** 10.3390/genes13122253

**Published:** 2022-11-30

**Authors:** Hongmei Shi, Jing Fu, Yang He, Zijian Li, Jiajia Kang, Changjie Hu, Xiannian Zi, Yong Liu, Jinbo Zhao, Tengfei Dou, Junjing Jia, Yong Duan, Kun Wang, Changrong Ge

**Affiliations:** 1 College of Animal Science and Technology, Yunnan Agricultural University, Kunming 650201, China; 2 Kunming Animal Health Supervision, 118 Gulou Road, Kunming 650223, China

**Keywords:** Tengchong snow, skeletal muscle development, muscle fibers, melanins

## Abstract

Tengchong snow, which has white feathers and black meat, is one of the most important black-bone chicken breeds and a genetic treasure of black food in China. Although the black meat traits are dominant, there are some chickens with white meat traits born in the process of folk selection and breeding. The purpose of this study was to compare the differences in skeletal muscle development between Tengchong snow black meat chickens (BS) and white meat chickens (WS), as well as whether excessive melanin deposition has an effect on skeletal muscle development. The BS and WS groups were selected to determine their muscle development difference at stages of 1, 7, 14, 21, and 42 days, using histological stain methods to analyze the development and composing type of breast and leg muscle fibers, as well as the count of melanin in BS muscle fibers. Finally, we were validated key candidate genes associated with muscle development and melanin synthesis. The results showed that BS breast muscle development was inhibited at 7, 14, and 21 days, while the leg muscle was inhibited at 7, 14, 21, and 42 days, compared to WS. Melanin deposition was present in a temporal migration pattern and was greater in the leg muscles than in the breast muscles, and it focused around blood vessels, as well as the epithelium, perimysium, endomysium, and connective tissue. Additionally, melanin produced an inhibitory effect similar to MSTN during skeletal muscle fiber development, and the inhibition was strongest at the stage of melanin entry between muscle fibers, but the precise mechanisms need to be confirmed. This study revealed that melanin has an inhibitory effect on the early development of skeletal muscle, which will provide new insights into the role of melanin in the black-boned chicken and theoretical references for the future conservation and utilization of black-boned chicken.

## 1. Introduction

The black-boned chicken with abnormal deposition of large amounts of melanin is an important genetic resource. Its main characteristics are “five blacks” (beak, skin, bones, legs, and flesh), which is also a distinctive economic characteristic to distinguish it from other chicken breeds [1]. In Asian countries, black-bone chicken is considered a nutritious food and has medical benefits, such as antioxidant activity, delaying aging, treating anemia, and the ability to treat menstrual abnormalities in women, and it is popular among consumers [2]. Moreover, the meat obtained from black-bone chickens is reported to contain lower amounts of fat and cholesterol than the meat of white chickens [3,4]. On the contrary, previous studies have found that the excessive melanin deposition in black-boned chickens inhibits its immune performance and reproductive performance [4,5]. However, there are no reports about the melanin’s effects on skeletal muscle development at present.

Growth performance and meat quality is the core of modern poultry breeding, and skeletal muscle, as the most vital component of the animal carcass, has a direct impact on the yield of poultry meat [6]. Muscle fibers are the basic units of skeletal muscle, and the amount of muscle in animals is mainly determined by the total number of muscle fibers (hyperplasia), thickness (hypertrophy), and types [7]. The number of muscle fibers is almost constant at birth in poultry, and the development of muscle after birth depends on the proliferation and differentiation of myoblasts and the function of myosatellite cells, which could promote myofiber hypertrophy and myofiber type conversion, leading to enlargement and thickening of skeletal muscle [8]. Hyperplasia occurs during the embryonic or early post-hatching period and is regulated by myogenic differentiation 1 (MYOD) [9]. Forced expression of MYOD is able to induce several cell lineages into myogenic cells, converting non-muscle cells (e.g., fibroblasts) into cells capable of fusing into myotubes and, eventually, fusing into myotubes [10]. It also has a response to muscle injury that is upregulated in activated satellite cells [11]. In addition, MYOD interacts with several related genes, such as Myf5, MEF2, and MRF4, to regulate muscle production and regeneration in the form of a gene network [8]. In contrast, myostatin (MSTN), also known as growth/differentiation factor-8 (GDF-8), is a TGF-superfamily member and a known negative regulator of muscle growth and development that is almost entirely expressed in skeletal muscle tissue [12,13]. Some studies have shown that MSTN KO chickens exhibit significantly larger skeletal muscles [6,14]. On the other hand, muscle development in poultry is significantly influenced by the metabolic properties (the type of muscle fibers). As compared to slow-growing poultry, fast-growing poultry had larger diameter fibers and a higher proportion of glycolytic fibers (fast-shrinking, glycolytic; type II fibers) [15,16]. Domestic chicken skeleton muscle is made up of various muscle fiber types (including type I and type II (IIa, IIb muscle fibers)) [17]. Type I corresponds to slow-contracting muscles, whereas types IIa and IIb correspond to fast-contracting muscles, and they are capable of interconversion during muscle growth. Additionally, the contractile properties of muscle fibers depend on the type of expressed myosin heavy chain (MyHC): MYH7B, slow-contracting myosin heavy chain (type I); MYH1A, fast-contracting myosin heavy chain (type IIB); MYH1B, fast-contracting myosin heavy chain (type IIA) [18].

The Tengchong snow is a unique local breed of black-boned chicken produced in Tengchong, a city in western China, and was identified during the 1995–1997 supplemental survey of livestock and poultry genetic resources [19]. It has medicinal materials and has been used as the main ingredient of Wu Ji Bai Feng Wan (gynecological medicine). According to the local records, the Tengchong snow was formed by the crossing of the grade II protected animal Tibetan snowcock (*Tetraogallus tibetanus*) with local domestic chickens in the Gaoligong Mountains [20]. Tengchong snow is covered with snowy white feathers in a medium-sized body. It grows fast and has black meat and bones. Because of its long period of free range, the Tengchong snow was able to adapt to its surroundings and develop a strong resistance to disease. In general, at sexual maturity, the female chicken weight of Tengchong snow is 1728.14 g, while the male chicken’s is 2083.48 g [21,22]. Additionally, the meat of Tengchong snow is found to have higher protein, minerals, and intramuscular fat, compared to other native chicken breeds [20,23]. However, due to long-term, free-range, and random mating, the Tengchong snow trait separated and formed the black meat traits with a small number (about 20%) of white meat traits. Black birds have black skin, muscle, bone, internal organs, and fat, but white birds had an absence of melanin in their cockscomb, claws, skin, and muscle. Currently, few studies have been reported on the differences in the skeletal muscle development status of the Tengchong snow with black meat or white meat.

The previous study found that the rapid growth period of Tengchong snow is 0–8 weeks, while the marketable age of modern broiler chickens is 6–7 weeks [24,25]. Therefore, this research was conducted to compare the differences in skeletal muscle during the early developmental stages (1 d, 7 d, 14 d, 21 d, and 42 d) of Tengchong snow from the black meat (BS) and white meat (WS) groups and to explore whether the deposition of melanin in black-bone chickens has an effect on the development of muscle fibers. This research will provide a scientific and theoretical basis for the development and utilization of the black-boned chicken.

## 2. Materials and Methods

### 2.1. Animal Experimentation Ethical Statement

All animal experimental procedures were approved and guided by the Yunnan Agricultural University Animal Care and Use Committee (approval ID: YAUACUC01, publication date: 10 July 2013).

### 2.2. Chicken, Diet and Housing

BS and WS were purchased from the Yunnan Agricultural University Chicken Farm. The experimental chickens were sourced from Tengchong snow Mountain Breeder Farm (Gaoligong Mountain Snow Mountain Chicken Development Co., Ltd., Baoshan, China), which were proven to be local Tengchong snow by genetic background investigation and have been bred and preserved for many generations. A total of 400 female chickens, including 1 d of age (200 from each genotype), were reared under standard conditions on a starter diet (period I: 20.0% CP and 13.02 MJ/kg ME) to 30 d of age. From 30 d of age onward, chickens were fed a regular diet (period II: 18.6% CP and 12.8 MJ/kg ME) to 42 days (week 6), and individuals within each genotype had the same genetic background. Diet content was consistent with the formulation to meet NRC 1994 and Chinese Chicken Feeding Standard recommendations. Individuals within each breed had the same genetic background. The compositions of diets details are shown in Table 1 and Table 2.

### 2.3. Slaughter Procedure and Sample Collecting

Thirty female chickens each from WS and BS with similar body weight were selected for slaughter at 1 d, 7 d, 14 d, 21 d, and 42 d, respectively. Feed and water were restricted for 16 h and 12 h before slaughter. Chickens were slaughtered by cervical dislocation, in accordance with the National Experimental Animal Slaughter Standard of China. After slaughter, the phenotypes related to muscle development were evaluated, and the measurement methods are listed in Table 2. 

The left breast muscle (pectoralis major muscle (PM)) was taken in the same position as the right leg muscle (gastrocnemius muscle (GAS)). All specimens were taken from the central portion of the widest part of the leg muscle and the ventral surface of the breast muscle (facing the skin), excluding the most superficial 3 mm. These samples were cut into 3 pieces (0.5 cm × 0.5 cm × 1.0 cm in size). The breast and leg muscles were stored in lyophilized tubes in liquid nitrogen at −80 °C for subsequent section preparation and total RNA extraction.

### 2.4. Histological Study

#### 2.4.1. Cryosectioning and Hematoxylin and Eosin Staining

The muscle sections were produced with the frozen sectioning technique in this experiment. In brief, an appropriate amount of optical cutting temperature compound (OCT) (Sakura Finetek, Tokyo, Japan) was first added to a 25 mm × 40 mm × 5 mm embedding box (Citotest, Jiangsu, China), then the muscle sample was placed, and OCT was continuously added until the sample was completely covered. The embedded samples were frozen with liquid nitrogen and fixed in the frozen sectioning machine (−25 °C) (Leica Biosystems, Wetzlar, Germany). Then, they were cut into 10-um flakes and transferred to glass slides before being stored at −20 °C until histological staining.

Muscle fiber morphological traits were determined using hematoxylin and eosin (H&E) staining. All staining reagents were purchased from Solarbio (Solarbio Life Science, Beijing, China), and the following programs were used: hematoxylin, 5 min; water washing, 10 min; 1% acid alcohol differentiation solution, 25 s; water washing, 15 min; eosin, 3 min; water washing, 2 min. After staining, the slices were dehydrated and transparently treated before being sealed with neutral resin. With the Nikon ECLIPSE TI-S image acquisition system (Nikon Corporation, Tokyo, Japan), the diameter, single cross-sectional area, and density of muscle fibers at 200× magnification were examined. The fiber diameter and cross-sectional area were calculated using an image analysis system (Image-Pro Plus, Media Cybernetics, Rockville, MD, USA), and density means the number of muscle fibers in 1 mm^2^. For each sample, 3 different points on 3 images containing approximately 300 muscle fibers were estimated.

#### 2.4.2. ATPase Staining

Muscle fiber type is determined synergistically by myosin ATPase. Myosin ATPase staining was referenced by Brooke and Kaiser [26] and Sen et al. [27], with appropriate adjustments. The following programs were used: Pre-incubation (pH = 4.3), 10 min at 37 °C; distilled water washing, 3 times and each 2 min; ATP-incubation (pH = 9.6), 45 min at 37 °C; 10% calcium chloride solution washing and incubate for 3 min; 2% cobalt nitrate solution, incubation 3 min at room temperature (22 °C); distilled water washing, 3 times and each 2 min; 1% ammonia sulfide solution, incubate for 3 min at room temperature; water washing, 5 min. The different types of muscle fibers were classified according to their color as oxidative fibers (darkness) and glycolytic fibers (lightness).

### 2.5. mRNA Expression Analysis

Seven candidate genes were selected for qRT-PCR validation. After extracting total RNA from breast muscle and leg muscle tissue, the total RNA reverse transcriptase kit (Takara, Kusatsu, Japan) was used to synthesize cDNA. Real-time PCR ABI 7500 Fast Real-Time PCR System using SYBR premix Ex TaqTM II (Takara, Kusatsu, Japan) was used to perform real-time PCR. The 2^−∆∆CT^ method was used to determine relative expression, and ACTB was used as the internal control for normalization of the results. The experiment was repeated in triplicate. All primer sequences were listed shown in Table 3.

### 2.6. Data and Statistical Analysis

All experimental data were evaluated as means ± standard error. All data were analyzed by two-way ANOVA to evaluate the main effect factors between genotype and days old in SPSS statistical software (version 18.0, SPSS, Chicago, IL, USA), and the differences between the two genotypes were compared by applying the parametric Tukey’s honestly significant difference (HSD) test. *p*-values of < 0.05 were considered statistically significant. All data visualization were performed using GraphPad Prism (version 9.4, GraphPad Software, San Diego, CA, USA).

## 3. Results

### 3.1. Comparative Analysis of Early Development in Skeletal Muscle

**Figure 1:**
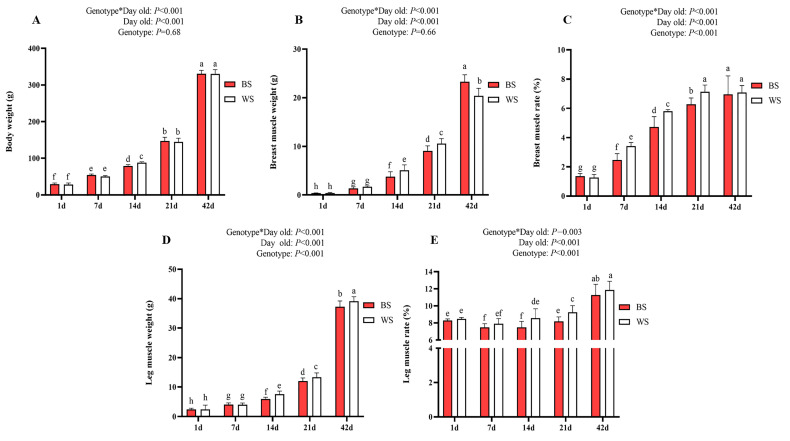
Comparative analysis of early development in skeletal muscle between BS and WS. (**A**) Comparison of body weight between two genotypes; (**B**) Comparison of breast muscle weight between two genotypes; (**C**) Comparison of breast muscle rate (breast muscle weight/body weight (%)) between two genotypes; (**D**) Comparison of leg muscle weight between two genotypes; (**E**) Comparison of leg muscle rate (leg muscle weight/body weight (%)) between two genotypes. Vertical bars represent mean ± SD (*n* = 30). Statistically significant differences are indicated by different letters (*p* < 0.05).

To compare the differences in early skeletal muscle development between BS and WS, we measured body weight, breast muscle weight, breast muscle rate, leg muscle weight, and leg muscle rate for different genotypes, shown in Figure 1. There were some significant interactions between days old and genotypes for body weight (*p* < 0.001, Figure 1A), breast muscle weight (*p* < 0.001, Figure 1B), breast muscle rate (*p* < 0.001, Figure 1C), leg muscle weight (*p* < 0.001, Figure 1D), and leg muscle rate (*p* < 0.01, Figure 1E). The comparison of body weight between BS and WS is shown in Figure 1A. The mean weight was not significantly different between the two groups, except on 14 d, where the mean weight of the BS was significantly lower than the WS (*p* < 0.05). The comparison of breast muscle weight between BS and WS is shown in Figure 1B. The breast muscle weight of the BS was significantly lower than the WS on 14 d and 21 d (*p* < 0.05), and the BS was significantly higher than the weight of the WS on 42d (*p* < 0.05). The breast muscle rate of the BS was significantly lower than the WS on 7 d, 14 d, and 21 d (*p* < 0.05, Figure 1C). The leg muscle weight of the BS was significantly lower than the WS on 14 d, 21 d, and 42 d (*p* < 0.05, Figure 1D). The leg muscle rate of the BS was significantly lower than the WS on 14 d and 21 d (*p* < 0.05, Figure 1E).

### 3.2. Comparative Analysis of Skeletal Muscle Fiber

#### 3.2.1. Comparative Analysis of Breast Muscle Fiber

**Figure 2:**
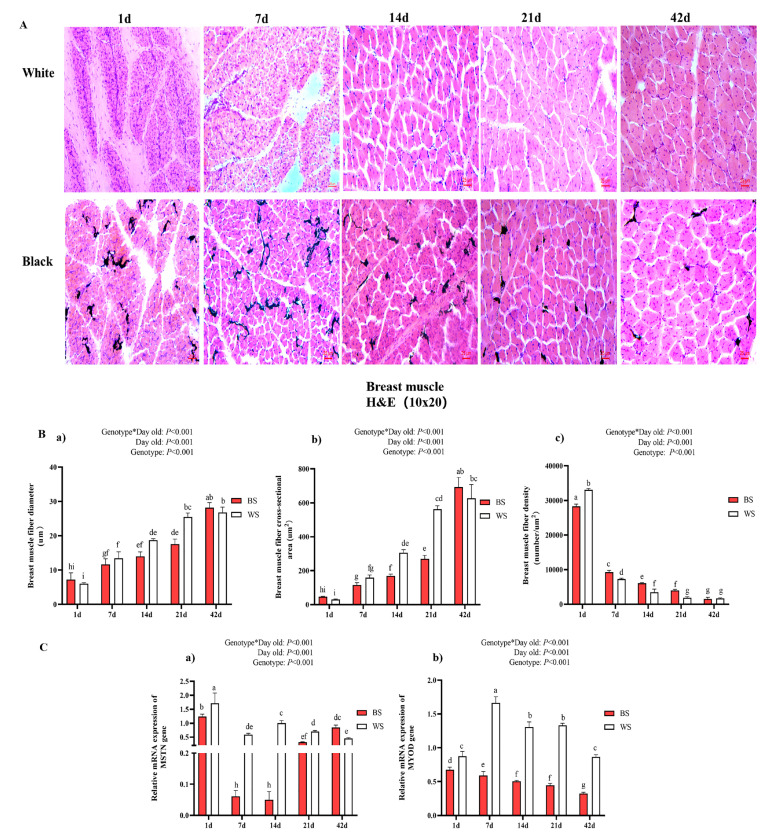
Comparative analysis of the histological stain of breast muscle fibers and related gene expression between BS and WS. (**A**) Histology of breast muscle fibers in 1–42 d; H&E stain (10 × 20). (**B**) Comparison of histological characteristics of breast muscle fiber of the two genotypes. (**a**) Muscle fiber diameter; (**b**) muscle fiber cross-sectional area; (**c**) fiber density. (**C**) Expression of MSTN gene (**a**) and MYOD gene (**b**) in BS and WS breast muscles. Vertical bars represent mean ± SD (*n* = 10). Statistically significant differences are indicated by different letters (*p* < 0.05).

To compare the breast muscle fiber traits and the expression levels of genes that are related to muscle development between BS and WS from 1 d to 42 d, we prepared frozen sections of two groups of breast muscles and analyzed the characteristics of their muscle fibers using HE staining. It was found that the morphology of muscle myofibrils in both was similar, predominantly elliptical in shape, and increased with age. However, a large amount of melanin was distributed in the BS muscle tissue (Figure 2A). Additionally, we did not know whether the distribution of melanin within the muscle would cause differences in myofiber characteristics between the black and white chickens’ breast muscles, and we proceeded to analyze their myofiber traits, including fiber diameter, cross-sectional area, and density. The diameter and single cross-sectional area of BS and WS breast muscle fibers increased with age, while density decreased. In addition, some significant main effects were observed for different ages and genotypes of the fiber diameter, cross-sectional area, and fiber density in breast muscles (Figure 2B). At 1 d, there was a significant difference (*p* < 0.05) in the density of muscle fibers between BS and WS, and BS was smaller than WS. During 7–21 d, the diameter and cross-sectional area of breast muscle fibers of WS were larger than those of BS, and the difference was significant (*p* < 0.05), while the density was the opposite. At 42 d, the BS muscle fiber cross-section was significantly larger than that of WS (*p* < 0.05), but their densities were not significantly different. Additionally, we examined the expression of genes, MSTN and MYOD, that are related to muscle development (Figure 2C). The expression trend of MSTN in the muscles of BS decreased, followed by an increasing trend, while the high level was almost always maintained in WS. WS had significantly higher MSTN expression than BS in both 1–21 d (*p* < 0.05), but BS had significantly higher MSTN expression than WS at 42 d (*p* < 0.05). The trend of MYOD expression within BS showed a continuous decrease, while WS showed an increase, followed by a decrease. The MYOD expression levels in WS were significantly higher than those in BS from 1–42 days (*p* < 0.05).

#### 3.2.2. Comparative Analysis of Leg Muscle Fiber

**Figure 3:**
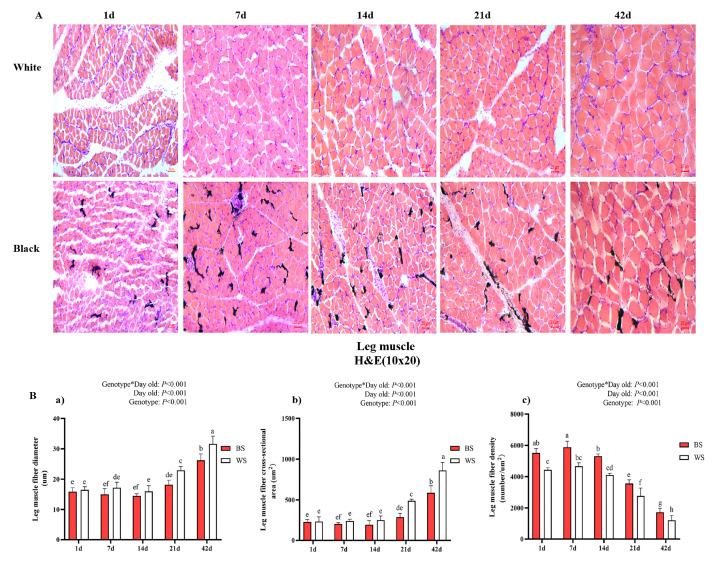

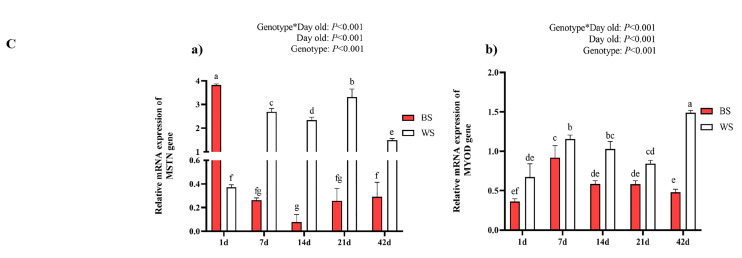
Comparative analysis of the histological stain of leg muscle fibers and related gene expression between BS and WS. (**A**) Histology of leg muscle fibers in 1–42 d; H&E stain (10 × 20). (**B**) Comparison of histological characteristics of leg muscle fiber of the two genotypes. (**a**) Muscle fiber diameter; (**b**) muscle fiber cross-sectional area; (**c**) fiber density. (**C**) Expression of MSTN gene (**a**) and MYOD gene (**b**) in BS and WS leg muscles. Vertical bars represent mean ± SD (*n* = 10). Statistically significant differences are indicated by different letters (*p* < 0.05).

To compare the morphology traits of leg muscle fibers in BS and WS, representative characteristics and expression levels of related genes were investigated (Figure 3). Additionally, we prepared frozen sections of two groups of leg muscles and found that the morphologies of muscle myofibrils in both were similar, predominantly elliptical in shape, and increased with age. Meanwhile, a large amount of melanin was distributed in the BS leg muscle tissue (Figure 3A). Next, we proceeded to analyze their myofiber characteristics, including diameter, cross-sectional area, and density, as shown in Figure 3B. There was significant interaction between factors (day old and genotype) for the fiber diameter, cross-sectional area, and fiber density of leg muscles. At 1 d, there was a significant difference (*p* < 0.05) in the density of muscle fibers between BS and WS, and BS was smaller than WS. At 7 d, BS had a significantly smaller leg muscle fiber diameter than WS (*p* < 0.05) and the opposite density, which was significantly larger than WS (*p* < 0.05). At 14 d, only the density was significantly different, and BS was larger than WS (*p* < 0.05). At 21 d and 42 d, the BS diameter and cross-sectional area were both significantly smaller than WS (*p* < 0.05), with the opposite density.

Lastly, we examined the expression of genes (MSTN and MYOD) related to muscle development (Figure 3C). The expression trend of MSTN in the leg muscles of BS showed a decreasing, and then increasing, trend, while the high level was almost always maintained in WS, except 1 d. BS had significantly higher MSTN expression than WS at 1 d (*p* < 0.05), but WS had significantly higher MSTN expression than WS from 7 d to 42 d (*p* < 0.05). MYOD expression in BS leg muscles increased from 1 d to 7 d, and it decreased and plateaued after 7 d, whereas it increased and then decreased in WS and increased again by 42 d. At all ages, the expression of MYOD was significantly lower in BS than in WS.

#### 3.2.3. Comparative Analysis of the Leg Muscle Fiber Types

**Figure 4:**
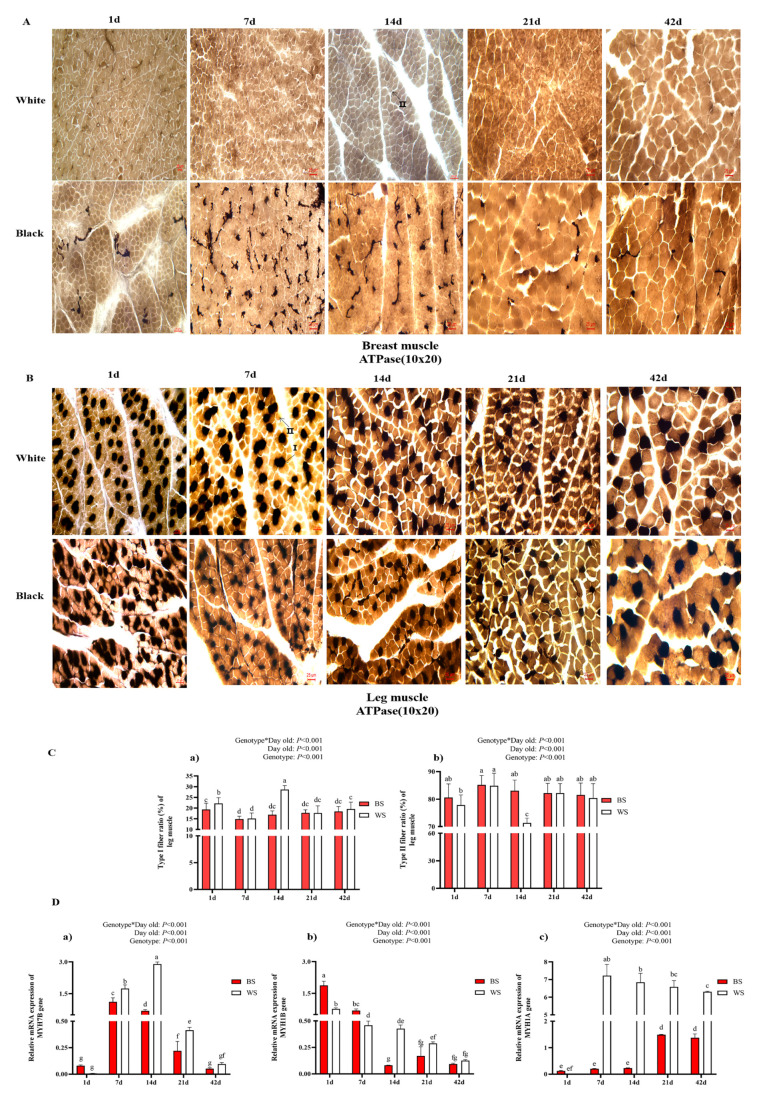
Comparative analysis of leg muscle fiber types and related gene expression between BS and WS. (**A**) Histology of breast muscle fibers at 1–42 d; ATPase staining (10 × 20). (**B**) Histology of leg muscle fibers at 1–42 d; ATPase staining (10 × 20). (**C**) Fiber types between the BS and WS leg muscle fibers: (**a**) ratio of type I fiber; (**b**) ratio of type II fiber. (**D**) Relative mRNA expression of MYHC isoform genes in BS and WS leg muscles; (**a**) MYH7B, (**b**) MYH1B, and (**c**) MYH1A. MYH7B, (type I) slow-twitch myosin heavy-chain; MYH1B, (type IIa) fast-twitch myosin heavy-chain; MYH1A, (type IIb) fast-twitch myosin heavy-chain. Vertical bars represent mean ± SD (*n* = 10). Statistically significant differences are indicated by different letters (*p* < 0.05).

To explore whether muscle fiber type was responsible for the differences in muscle development between BS and WS in early development, we performed ATPase acid staining (pH = 4.3) on frozen sections of muscle, and the results of the ATPase staining of breast muscle are shown in Figure 4A, with only type II and no type I muscle fibers at all day ages. A small amount of type I (dark staining) with a large amount of type II (light staining) was present in the leg muscles (Figure 4B). From the type I ratio and type II ratio in the leg muscles of BS and WS, there was a significant interaction between factors (day old and genotype) for leg muscle fiber types and the related gene expression. It can be shown that the type I ratio of leg muscle fibers in BS is lower than in WS at all ages, but has a significant difference only at 14 d, and the type I ratio was significantly lower in BS than in WS (*p* < 0.05, Figure 4C), while the type II ratio was the opposite. Then, the expression of MYHC isoform genes (MYH7B, MYH1B, and MYH1B) that determine muscle fiber type in BS and WS leg muscles were detected by qRT-PCR (Figure 4D). The expression of MYH7B (type I) in BS leg muscle was significantly lower than WS at 7 d, 14 d, and 21 d (*p* < 0.05). MYH1B (type IIa) in BS was significantly higher than WS at 1 d and 7 d and significantly lower than WS at 14 d and 21 d (*p* < 0.05). MYH1A (type IIb) in BS was significantly lower than WS at 7 d, 14 d, 21 d, and 42 d (*p* < 0.05). In general, the expression of MYH7B (type I) and MYH1A (type IIb) in leg muscles is lower in BS than in WS.

#### 3.2.4. Melanin Distribution of the Breast and Leg Muscles in BS

**Figure 5:**
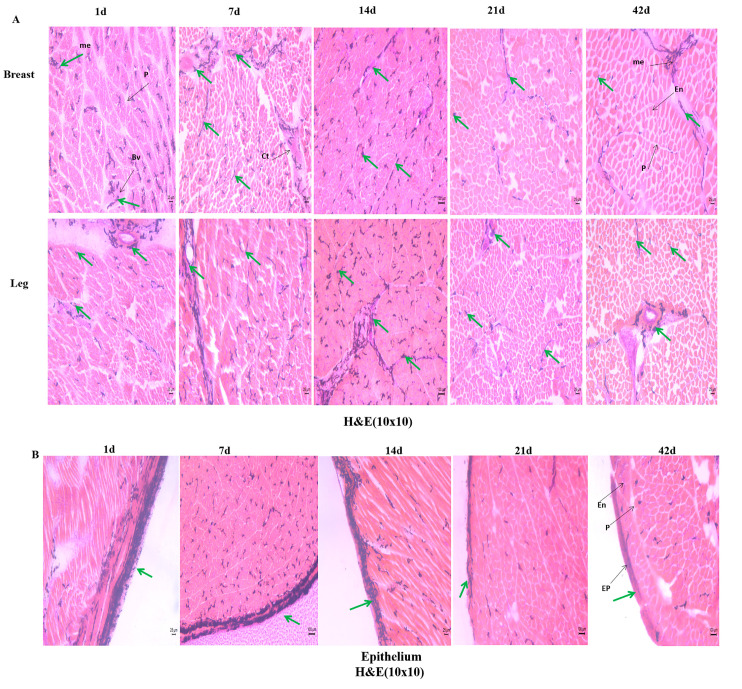

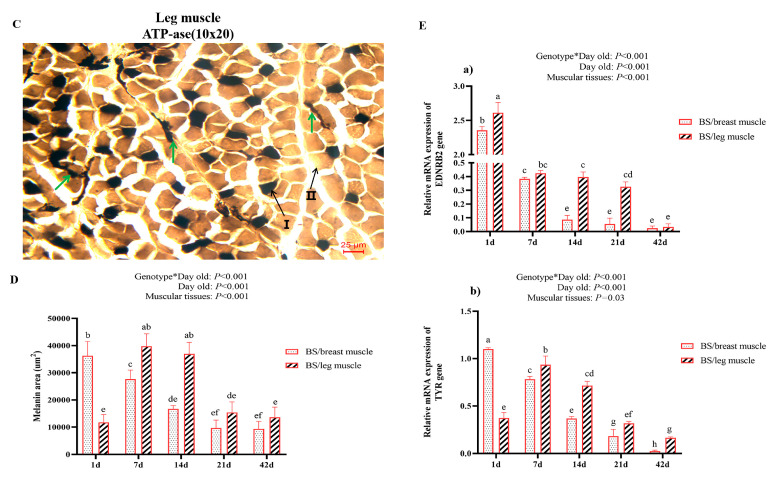
Melanin distribution and related gene expression of the BS. (**A**) Melanin (indicated by green arrows) distribution among the breast and leg muscle fibers in BS and mainly deposited in the epithelium, perimysium, endomysium, connective tissue, and around blood vessels; H&E stain (10 × 10); EP: epithelium, P: perimysium, En: endomysium, Bv: blood vessel, Ct: connective tissue. (**B**) Melanin distribution on the myoepithelium; H&E stain (10 × 10). (**C**) The distribution of melanin between type I and type II muscle fibers; ATPase stain (10 × 20). (**D**) The amount of melanin in the muscle fibers of the breast and leg muscles in BS. (**E**) EDNRB2 (**a**) and TYR (**b**) gene expression in BS breast and leg muscles. Vertical bars represent mean ± SD (*n* = 10). Statistically significant differences are indicated by different letters (*p* < 0.05).

There was abnormal melanin deposition in the BS, which led to the color of the breast and leg muscles appearing gray or gray-black. The deposition of melanin in the muscle is shown in Figure 5A–C. Melanin was mainly deposited in the epithelium, perimysium, endomysium, connective tissue, and around blood vessels (Figure 5A). In the perimysium, melanin is fibrillar or dendritic, and closer to the muscle fibers. In the endomysium, melanin is sporadically distributed in a linear pattern. The distribution of melanin in the breast muscles is more fragmented than in the leg muscles, and the amount of melanin deposited in the muscle fiber is less than in the leg muscles. In the epithelium, compared with other locations, the melanin deposition was more and tightly concentrated, and it was gradually less with age, especially after 14 d (Figure 5B). Additionally, the majority of the melanin in the muscle fibers was found near type II fibers, with only a few melanin found near type I fibers (Figure 5C). With the growth, melanin in BS muscle tissue is migrated and deposited in a certain order: at 1 d, melanin is deposited mainly in the epithelium, perimysium, connective tissue, and around blood vessels, in large quantities and with a dense distribution; melanin moved from epithelium, perimysium, connective tissue to endomysium at 7 d, 14 d, and 21 d. The amount of melanin in endomysium increased by a lot, and the distribution became more scattered; by 42 d, the overall number of myofiber migrated from endomysium to the epithelium, perimysium, and connective tissue, reaching a minimum compared to other days.

According to the statistical results of melanin between muscle fibers in Figure 5D. There was significant interaction between factors (day old and muscular tissues) for the melanin content and related gene expression. The content of melanin in BS breast muscles gradually decreased with age; the melanin content in the leg muscles increased before 7 d and started to decrease gradually after 7 d. Melanin was significantly lower in the breast muscles than in the leg muscles at all ages, except 1 d and 42 d in BS (*p* < 0.05). Moreover, we tested the expression of genes related to melanin production and deposition, including the EDNBR2 gene, which mediates melanocyte migration, and the TYR gene, a key enzyme for melanin synthesis. Both were not expressed in the breast and leg muscles of WS, and their expression in BS is shown in Figure 5E. The expression of the EDNRB2 gene in both BS breast and leg muscles tended to decrease gradually, and at 1 d, 14 d, and 21 d, the expression in breast muscles was significantly lower than that in leg muscles (*p* < 0.05). In the BS breast muscle, the expression of the TYR gene kept decreasing with age, while in the leg muscle, it increased before 7 d and gradually decreased after 7 d. At 1 d, the expression of TYR in the breast muscle was extremely significantly higher than that in the leg muscle (*p* < 0.05). In contrast, from 7 d to 42 d, it was significantly higher in the leg muscle than in the breast muscle (*p* < 0.05).

## 4. Discussion

Melanin content is considered to be the most important indicator of the meat quality of black-boned chicken, providing consumers with the first visual and directly determining their purchase decision. Referring to the previous study [28], we used tissue staining to calculate the melanin content between muscle fibers and found that the content of melanin, except for 1d in the leg muscle fibers, was greater than the breast muscles in the BS. Additionally, the content of melanin gradually decreased with the increase of age. Previous research in black-bone chickens discovered that muscle contraction was caused by the inward movement of Ca^2+^ in the extracellular matrix and led to pigment granule aggregation. The pigment granules are primarily found in locomotion muscles, such as the leg muscles, which are typically darker in color than breast muscles [28,29]. The melanin in BS muscle fibers was primarily distributed around blood vessels, as well as the epithelium, perimysium, endomysium, and connective tissue, which was consistent with the pattern of melanin deposition in silky fowl and Thai native black-bone chickens [5,29]. In addition, melanin-based pigmentation is a complex process that includes the migration of melanocytes and the synthesis of melanin. EDNRB2 expression in melanocytes is to support their migration by overcoming repulsive or non-permissive cues in the dorsolateral environment [30]. In poultry, changes in the expression of EDNRB2 resulted in the impaired migration of NCCs, and mutations in EDNRB2 result in white plumage coloration in Chinese geese [30]. The mRNA expression level of EDNRB2 increases with the expansion of the melanin region on the body surface feathers of duck embryos [31]. Hence, we identified melanocytes in skeletal muscle by determining EDNRB2 expression. The amount of melanin in muscle fibers gradually decreased with age, and the expression of EDNRB2 mRNA in breast muscle was consistent with it. However, there was an inconsistency between EDNRB2 expression and melanin content in the leg muscles. Analysis in the sections showed that melanin was most distributed in the leg muscles at 7 d and 14 d, but EDNRB2 expression was low in these periods. Since melanin content also correlates with the ability of melanin to be synthesized by melanocytes, in which the process is regulated by the TYR, we went on to measure muscle melanin synthesis by assessing TYR expression levels. The expression of TYR mRNA in the leg muscles showed higher levels at 7 d and 14 d. Therefore, we suggest that the difference in melanin synthesis capacity is responsible for the difference in melanin content between the breast and leg muscles during this period.

In this study, BS weight was inhibited at 14 d, and breast muscle development (breast muscle weight, breast muscle rate, and muscle fibers traits) was also inhibited at 7 d, 14 d, and 21 d. The leg muscle development (leg muscle weight, leg muscle rate, and muscle fibers traits) was also inhibited at 7 d, 14 d, 21 d, and 42 d. Similarly, at these ages, the melanin content in the muscle fibers was high and was deposited in large amounts in the endomysium. Previous studies have found that excessive melanin deposition in black-boned chickens inhibits immune performance and reproductive performance [5]. Hence, we hypothesized that the inhibition of breast and leg muscles development is caused by the production of large amounts of melanin at these stages, as well as the entry of melanin between muscle fibers. The inhibition of BS breast muscle was primarily concentrated at 7 d, 14 d, and 21 d, and it recovered after 21 d, whereas the inhibition of leg muscle began at 7 d and did not end until 42 d, and the melanin deposition in leg muscle was greater than that in breast muscle. Therefore, we believe that differences in the site and time of deposition of melanin are one of the reasons for the more persistent inhibition in the leg muscles than in the breasts.

Early myofiber hypertrophy in chicks is mainly driven by the fusion of mononuclear myogenic stem cells (MSCs, also known as satellite cells) with existing myofibers and the deposition of myogenic fibronectin, resulting in myofibers with more nuclei and a larger cross-sectional area (CSA) [32]. In breast and leg muscle development, the expression of both the muscle development-related negative regulator MSTN (which negatively regulates muscle development through cell differentiation in embryonic somites and myogenic fiber growth in adulthood [33]), and the positive regulator MYOD (MyoD induces fibroblasts into myogenic cells, which then differentiate and fuse into myotubes [34]) was significantly lower in BS than in WS (*p* < 0.05). Therefore, we presume that the synthesis of melanin inhibits muscle development and the expression of related regulatory factors. In particular, the MSTN gene, a repressor of muscle development, was also expressed at a lower level in BS than in WS. In fact, the low expression of MSTN in BS should promote muscle development over WS, but muscle development in BS was also inhibited during this period. We hypothesized that massive melanin has the same effect as MSTN in inhibiting muscle development. Previous studies found an approximately 30% increase in MyoD+ myogenic stem cell (MSC) populations in fast-growing ROSS broiler chickens, compared with slow-growing RR birds [32]. Our results show that WS had significantly higher MYOD expression than BS, due to the lack of melanin synthesis. Similarly, MYOD gene expression in muscles followed the same trend as melanin content in muscle fibers, with MYOD consistently decreasing from 1 d in BS breast muscles and from 7 d in leg muscles. As a consequence, we assume that the massive synthesis of melanin consumes a lot of energy in BS, resulting in the suppression of the expression of both positive and negative regulatory factors acting on muscle development, ultimately leading to skeletal muscle development suppression.

The development of skeletal muscle is also affected by the composition of muscle fiber types. Hence, we also examined the ratio of fast muscle fibers (type II) and slow muscle fibers (type I), as well as the expression of myosin heavy chain-related genes (MYH1A, MYH1B, and MYH7B) in the leg muscles of two groups. The results showed that the breast muscle of both chickens was composed of fast muscle fibers, and the leg muscles of WS and BS were composed both of fast muscle and slow muscle fibers. This finding is consistent with previous studies, that the pectoralis major muscle in 1-day-old snow mountain chickens (slow-growing broilers), and Ross 308 broilers (fast-growing broilers) consisted of type IIB fibers only, and a few types I fibers (about 17.55%) were identified in the gastrocnemius muscle of Ross 308 broilers [16]. In addition, BS possessed a higher percentage of type II than WS during leg muscle development, but the expression of related genes could not be matched fully. This finding is different from the reports that the expression of MYH1B (type IIA), MYH1A (type IIB), and MYH7B (type I) in the duck [35]. We assumed there may be three factors underlying this. Firstly, due to the limitations of ATPase stains, it was hard to distinguish the muscle fibers of types IIA and IIB, so their percentage was not consistent with the expression of the gene. Secondly, the genetic background and species is differently. Finally, the synthesis of melanin may influence the transform of muscle fibers. At last, on 14 d of BS muscle development, the expression of TYR in the BS is high, which could lead to an inhibition of the transformation of type I muscle fibers to type II, but this needs further verification.

## 5. Conclusions

In this study, differences in skeletal muscle development between BS and WS at early developmental stages (1 d, 7 d, 14 d, 21 d, 42 d) were assessed by comparing breast and leg muscle weight, breast and leg muscle rate, muscle fiber diameter, cross-sectional area, density, and the ratio of muscle fiber type I and type II. The results revealed that the development of BS’s breast muscles and leg muscles was inhibited, while the depositing of melanin may be the major reason. The development of the leg muscles was inhibited strongly and for longer, due to the higher melanin content than the breast muscles. It was also shown that melanin had an inhibitory effect similar to MSTN, and that the inhibitory effect was strongest when the melanin entered the muscle fiber deposition stage. Our results first demonstrated that melanin has an inhibitory effect on the early development of skeletal muscle, which provides new insights into the role of melanin in the black-boned chicken and will serve as a theoretical reference for the black-boned chicken’s future conservation and utilization.

## Figures and Tables

**Table 1:** Dietary composition and nutrient levels.

Items (Unit)	Period I	Period II
Corn (%)	63.25	60.19
Soybean meal (%)	30.26	25.88
Wheat bran (%)	0.00	10.00
Fish meal (%)	2.50	0.00
CaCO_3_ (%)	1.11	1.06
CaHPO_4_ (%)	1.50	1.50
Met (%)	0.08	0.07
NaCl (%)	0.30	0.30
Premix (%)	1.00	1.00
ME (MJ/kg^−1^)	13.02	12.8
CP (%)	20.00	18.60

Note: Main composition of premix (converted to ration per kg): VA 15,000 U, VE 62.5 mg, VK 3.6 mg, VBl 3 mg, VB 2 9 mg, VB 6 6 mg, Cu 12 mg, Zn 75 mg, Mn 60 mg, I 0.35 mg, Se 0.15 mg, nicotinamide 60 mg, Dpantothenic acid 18 mg, folic acid 1.5 mg, biotin 0.36 mg, VB 12 0.03 mg, Fe 80 mg, choline chloride 600 mg, antibacterial growth promoters, antioxidants.

**Table 2:** The measurement of phenotypes.

Phenotypes	Measurement
Body weight	The weight of bird before slaughter
Breast muscle weight	The weight of the breast without skin and adherent fat
Breast muscle rate	Percentage of breast muscle weight in body weight
Leg muscle weight	The weight of the two legs without skin and adherent fat
Leg muscle rate	Percentage of leg muscle weight in body weight

**Table 3:** qPCR primer information.

Gene	GenBank Accession	Primer Sequence (5′-3′)	Length (bp)	Product Size (bp)	Annealing Temperature (°C)
*ACTB*	NM_205518.2	F: TGAGCGCAAGTACTCTGTCTG	21	154	61.7
		R: TGTGGGTGTTGGTAACAGTCC	21		
*MSTN*	NM_001001461.2	F: AAACGGTCCCGCAGAGATTT	20	195	61
		R: CAGGTGAGTGTGCGGGTATT	20		
*MYOD*	NM_204214.3	F: ACTACAGCGGGGAGTCAGAT	20	190	61
		R: ATGCTTGAGAGGCAGTCGAG	20		
*MYH7B*	NM_204587.4	F: TGACAACGCCTACAACGACA	20	154	61.7
		R: TACTTTCTTGCCCGGTGTGT	20		
*MYH1B*	NM_204228.4	F: GGTCAACAAGCTCCGAGCAA	20	200	61.7
		R:TGCTGTATATGCAGAGAATCTATG	25		
*MYH1A*	NM_001013396.1	F: CTTCCAGTCAGCACAAGACCT	21	168	61.7
		R: AAGGCTTATTCTGGGCCTCG	20		
*EDNRB2*	NM_204120.1	F:AGAGGGACAACACAAAAGTAAAC	25	158	61
		R: GCCAGGTTCCTAGTGAATTAAACTT	25		
*TYR*	NM_204160.1	F: TGCTCAGATGAACAACGGCT	20	179	61.7
		R: GCAGAAAAGCACGATGCCAA	20		

Note: All the primers are designed with Primer 5.0 software and synthesized by Nanjing Qingke bioengineering company. F, forward primer. R, reverse primer.

## Data Availability

The data presented in this study are available on request from the corresponding author. The data are not publicly available due to privacy.

## References

[1] Li D., Sun G., Zhang M., Cao Y., Zhang C., Fu Y., Li F., Li G., Jiang R., Han R. (2020). Breeding history and candidate genes responsible for black skin of Xichuan black-bone chicken. BMC Genom..

[2] Yu S., Wang G., Liao J., Tang M. (2018). Transcriptome profile analysis identifies candidate genes for the melanin pigmentation of breast muscle in Muchuan black-boned chicken. Poult. Sci..

[3] Jaturasitha S., Srikanchai T., Kreuzer M., Wicke M. (2008). Differences in Carcass and Meat Characteristics Between Chicken Indigenous to Northern Thailand (Black-Boned and Thai Native) and Imported Extensive Breeds (Bresse and Rhode Island Red). Poult. Sci..

[4] Tian Y., Sheng Z., Xie M., Wang W., Wu H., Gong D. (2011). Composition of fatty acids in the muscle of black-bone silky chicken (*Gallus gellus demesticus brissen*) and its bioactivity in mice. Food Chem..

[5] Han D., Wang S., Hu Y., Zhang Y., Dong X., Yang Z., Wang J., Li J., Deng X. (2015). Hyperpigmentation Results in Aberrant Immune Development in Silky Fowl (*Gallus gallus domesticus* Brisson). PLoS ONE.

[6] Xu K., Zhou H., Han C., Xu Z., Ding J., Zhu J., Qin C., Luo H., Chen K., Jiang S. (2021). Transcriptomic Analysis of MSTN Knockout in the Early Differentiation of Chicken Fetal Myoblasts. Genes.

[7] Muroya S., Tanabe R., Nakajima I., Chikuni K. (2000). Molecular characteristics and site specific distribution of the pigment of the silky fowl. J. Vet. Med. Sci..

[8] Ren L., Liu A., Wang Q., Wang H., Dong D., Liu L. (2021). Transcriptome analysis of embryonic muscle development in Chengkou Mountain Chicken. BMC Genom..

[9] Ding S., Nie Y., Zhang X., Liu X., Wang C., Yuan R., Chen K., Zhu Q., Cai S., Fang Y. (2019). The SNPs in myoD gene from normal muscle developing individuals have no effect on muscle mass. BMC Genet..

[10] Yang Z.Q., Qing Y., Zhu Q., Zhao X.L., Wang Y., Li D.Y., Liu Y.P., Yin H.D. (2015). Genetic effects of polymorphisms in myogenic regulatory factors on chicken muscle fiber traits. Asian-Australas. J. Anim. Sci..

[11] Yamamoto M., Legendre N.P., Biswas A.A., Lawton A., Yamamoto S., Tajbakhsh S., Kardon G., Goldhamer D.J. (2018). Loss of MyoD and Myf5 in Skeletal Muscle Stem Cells Results in Altered Myogenic Programming and Failed Regeneration. Stem Cell Rep..

[12] Xu K., Han C.X., Zhou H., Ding J.M., Xu Z., Yang L.Y., He C., Akinyemi F., Zheng Y.M., Qin C. (2020). Effective MSTN Gene Knockout by AdV-Delivered CRISPR/Cas9 in Postnatal Chick Leg Muscle. Int. J. Mol. Sci..

[13] Kim Y.S., Bobbili N.K., Paek K.S., Jin H.J. (2006). Production of a monoclonal anti-myostatin antibody and the effects of in ovo administration of the antibody on posthatch broiler growth and muscle mass. Poult. Sci..

[14] Kim G.D., Lee J.H., Song S., Kim S.W., Han J.S., Shin S.P., Park B.C., Park T.S. (2020). Generation of myostatin-knockout chickens mediated by D10A-Cas9 nickase. FASEB J..

[15] Du Y.F., Ding Q.L., Li Y.M., Fang W.R. (2017). Identification of Differentially Expressed Genes and Pathways for Myofiber Characteristics in Soleus Muscles between Chicken Breeds Differing in Meat Quality. Anim. Biotechnol..

[16] Huo W., Weng K., Li Y., Zhang Y., Zhang Y., Xu Q., Chen G. (2022). Comparison of muscle fiber characteristics and glycolytic potential between slow- and fast-growing broilers. Poult. Sci..

[17] Yu J.A., Wang Z., Yang X., Ma M., Li Z., Nie Q. (2021). LncRNA-FKBP1C regulates muscle fiber type switching by affecting the stability of MYH1B. Cell Death Discov..

[18] Zeng Y.T., Wang C., Zhang Y., Xu L., Zhou G.B., Zeng C.J., Zuo Z.C., Song T.Z., Zhu Q., Yin H.D. (2020). Improvac immunocastration affects the development of thigh muscles but not pectoral muscles in male chickens. Poult. Sci..

[19] Zhao T.J. (2018). A Preliminary Study on Breed Preservation and Development of Tengchong Snow Chicken. China Anim. Ind..

[20] Ou C.H., Li R.Q., Ding Z.B., Ye S.H., Duan G. (1999). Nutrient composition analysis of Tengchong snow meat at different ages and sexes. J. Southwest Minzu Univ. Nat. Sci. Ed..

[21] Wang R.Q., Zhang Z.F., Huang J., Qian L.D., Teng X.H., Miao Y.W. (2021). Genetic Diversity of mtDNA D-loop Region in Tengchong White Chicken. J. Yunnan Agric. Univ. Nat. Sci..

[22] Liao G.Z., Wang G.Y., Cheng Z.B., Jia J.J., Ge C.R. (2013). Proteomic Analysis of Tengchong Snowcock Muscles in Yunnan. Meat Res..

[23] Dong S.Y., Guo L., Gu D.H., Wang X.R., Kan H. (2018). Study on decocting technology and nutritional component analysis of Tengchong snow cock muscle. Meat Ind..

[24] Liu S.Y., Xiao X.X., Ying H., Miao Y.W., Ye S.H. (2016). Analysis and fitting of the growth curve of Tengchong snowcock. China Poult..

[25] Richard V.N.D., Susan J.L., Max F.R., Michael E.P., Chris M.A., Carl J.S. (2017). Transcriptome analysis of post-hatch breast muscle in legacy and modern broiler chickens reveals enrichment of several regulators of myogenic growth. PLoS ONE.

[26] Brooke M.H., Kaiser K.K. (1970). Muscle fiber types: How many and what kind?. Arch. Neurol..

[27] Sen U., Sirin E., Ensoy U., Aksoy Y., Ulutas Z., Kuran M. (2016). The effect of maternal nutrition level during mid-gestation on postnatal muscle fibre composition and meat quality in lambs. Anim. Prod. Sci..

[28] Nganvongpanit K., Kaewkumpai P., Kochagul V., Pringproa K., Punyapornwithaya V., Mekchay S. (2020). Distribution of Melanin Pigmentation in 33 Organs of Thai Black-Bone Chickens (*Gallus gallus domesticus*). Animals.

[29] Kriangwanich W., Piboon P., Sakorn W., Buddhachat K., Kochagul V., Pringproa K., Mekchay S., Nganvongpanit K. (2021). Consistency of dark skeletal muscles in Thai native black-bone chickens (*Gallus gallus domesticus*). PeerJ.

[30] Harris M.L., Hall R., Erickson C.A. (2008). Directing pathfinding along the dorsolateral path—The role of EDNRB2 and EphB2 in overcoming inhibition. Development.

[31] Xi Y., Xu Q., Huang Q., Ma S., Wang Y., Han C., Zhang R., Wang J., Liu H., Li L. (2021). Genome-wide association analysis reveals that EDNRB2 causes a dose-dependent loss of pigmentation in ducks. BMC Genom..

[32] Tejeda O.J., Calderon A.J., Arana J.A., Meloche K.J., Starkey J.D. (2019). Broiler chicken myofiber morphometrics and myogenic stem cell population heterogeneity1,2. Poult. Sci..

[33] Bhattacharya T.K., Shukla R., Chatterjee R.N., Bhanja S.K. (2019). Comparative analysis of silencing expression of myostatin (MSTN) and its two receptors (ACVR2A and ACVR2B) genes affecting growth traits in knock down chicken. Sci. Rep..

[34] Tapscott S.J., Davis R.L., Thayer M.J., Cheng P.F., Weintraub H., Lassar A.B. (1988). MyoD1: A nuclear phosphoprotein requiring a Myc homology region to convert fibroblasts to myoblasts. Science.

[35] Huo W., Weng K., Gu T., Zhang Y., Zhang Y., Chen G., Xu Q. (2021). Effect of muscle fiber characteristics on meat quality in fast- and slow-growing ducks. Poult. Sci..

